# Endemic, cosmopolitan, and generalist taxa and their habitat affinities within a coastal marine microbiome

**DOI:** 10.1038/s41598-024-69991-3

**Published:** 2024-09-28

**Authors:** Chase C. James, Andrew E. Allen, Robert H. Lampe, Ariel Rabines, Andrew D. Barton

**Affiliations:** 1 Scripps Institution of Oceanography, University of California San Diego, 9500 Gilman Dr, La Jolla, CA 92093 USA; 2 University of Southern California, 3620 S Vermont Ave, Los Angeles, CA 90007 USA; 3 J. Craig Venter Institute, 4120 Capricorn Lane, La Jolla, CA 92037 USA; 4 Department of Ecology, Behavior and Evolution, University of California San Diego, 9500 Gilman Dr, La Jolla, CA 92093 USA

**Keywords:** Biogeography, Microbial ecology, Microbiome, Marine biology

## Abstract

The relative prevalence of endemic and cosmopolitan biogeographic ranges in marine microbes, and the factors that shape these patterns, are not well known. Using prokaryotic and eukaryotic amplicon sequence data spanning 445 near-surface samples in the Southern California Current region from 2014 to 2020, we quantified the proportion of taxa exhibiting endemic, cosmopolitan, and generalist distributions in this region. Using in-situ data on temperature, salinity, and nitrogen, we categorized oceanic habitats that were internally consistent but whose location varied over time. In this context, we defined cosmopolitan taxa as those that appeared in all regional habitats and endemics as taxa that only appeared in one habitat. Generalists were defined as taxa occupying more than one but not all habitats. We also quantified each taxon’s habitat affinity, defined as habitats where taxa were significantly more abundant than expected. Approximately 20% of taxa exhibited endemic ranges, while around 30% exhibited cosmopolitan ranges. Most microbial taxa (50.3%) were generalists. Many of these taxa had no habitat affinity (> 70%) and were relatively rare. Our results for this region show that, like terrestrial systems and for metazoans, cosmopolitan and endemic biogeographies are common, but with the addition of a large number of taxa that are rare and randomly distributed.

## Introduction

Marine microbes perform many ecosystem functions including primary production, nutrient cycling, and carbon sequestration^1–3^. These processes and their magnitudes depend upon microbial community composition, which is shaped by the spatial and temporal distribution of species^4,5^.

Like metazoans, marine microbial species distributions range from cosmopolitan, meaning a species is found in all or nearly all habitats, to endemic, meaning a species is found in one habitat, often with a relatively small area or restricted set of environmental conditions^6–8^. The high population sizes, rapid growth rates, and high dispersal abilities tied to the small body size of microbes suggests that cosmopolitan distributions should be fairly common^7,9,10^. Marine microbes disperse via water, air, biotic, or anthropogenic means, such that dispersal barriers, particularly for the smallest sizes, may be weak^11–14^. For example, the SAR11 Clade of bacteria has extremely large population sizes and has been found in nearly all marine habitats^15,16^. However, even with strong dispersal, environmental selection causes variation in community composition between similar habitats both within and between ocean basins^14^. In the marine environment, evidence is accumulating that marine microbes exhibit strong biogeographic patterns and endemism^17^. For example, Malviya et al. found that for marine diatoms, the percentage of endemic species within a region ranges from 2.3 to 53.3% of the observed diatom taxa depending on the region^18^. For ciliates, studies have observed that the percentage of endemic taxa within a region ranges from 8.8 to 16% of the total observed regional ciliate richness^19,20^. Even when marine microbes can disperse widely, strong selection, mutations, and demographic stochasticity allow for endemism and regional variations in community structure across similar environmental conditions^14^.

To date, and unlike for larger organisms^21^ and terrestrial systems^8^, no study of the marine microbiome has attempted to quantify the relatively frequency of endemic and cosmopolitan ranges among marine microbes. Nor do we have a clear understanding of the degree to which marine microbes are found across multiple, internally-consistent ocean habitats, or if they are preferentially found in a subset of habitats, while rarely found in others. A major reason for this knowledge gap is due to the difficulty of collecting observations repeatedly across large areas within the pelagic ocean. Another complication is that, unlike terrestrial systems, pelagic habitats, or seascapes, are defined by their physical and chemical characteristics, and move through time^22,23^. Thus, in order to assess microbial endemism and habitat specificity, it is necessary to capture pelagic habitats as they expand and contract seasonally and interannually^24–26^, and assess the frequency of occurrence of taxa within these shifting habitats.

**Figure 1:**
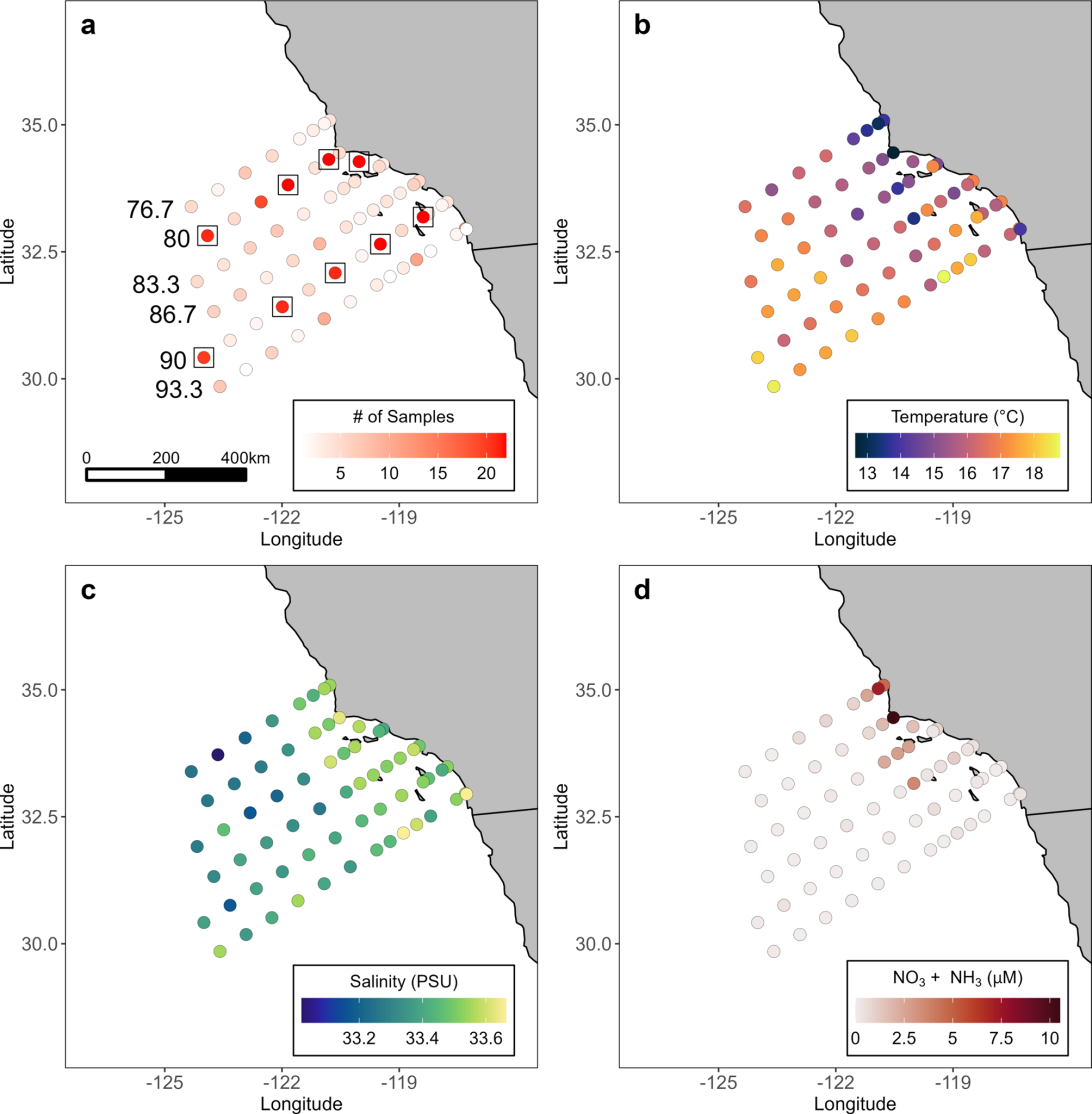
Description of the sampling regime and environment over 2014–2020. (**a**) Total number of samples per station. Squares highlight cardinal stations which are sampled every cruise. (**b**) Mean temperature (°C), (**c**) salinity (PSU), (**d**) and NO_3_ + NH_3_, µM per station across all cruises.

In this study, we adopted this pelagic habitat framework to ask the following questions: (1) are marine microbes found in preferred habitats and (2) what proportion of microbes are endemic and cosmopolitan within a regional context? We used a 7-year survey of marine microbial community composition consisting of 445 surface samples within the Southern California Current (SCC) region, referred to as the NOAA-CalCOFI Ocean Genomics, or NCOG, data^27^. This study includes 13,558 distinct bacteria/archaea (16S) amplicon sequence variants (ASVs) and utilized both 18S-V4 (21,095 ASVs) and 18S-V9 (25,815 ASVs) to characterize the eukaryotic community. The region is characterized by strong environmental gradients, from the highly productive nearshore to the oligotrophic offshore (Fig. 1). To explore these questions in a way that was comparable to terrestrial systems, we identified three internally consistent habitats within the SCC region, based upon temperature, salinity, and dissolved nitrogen (NH_3_ + NO_3_). These habitats change in time and space but are comparable to fixed-location terrestrial habitats in that their internal environments are relatively consistent. Following the classification of our samples into pelagic habitats, we identified the prevalence of regional endemism (found in one pelagic habitat) and cosmopolitanism (found in every pelagic habitat in this region). We also assessed what proportion of species had a significant affinity to zero, one, or two habitats within the region—testing whether selection for preferred habitats is a strong driver of regional microbial biogeographies. Here, affinity is defined as an ASV being significantly more abundant in a pelagic habitat relative to the null expectation (randomly distributed). Finally, we examined to what extent taxa found in NCOG were found elsewhere from data collected by *Tara* Oceans, *Tara* Polar, and BioGEOTRACES surveys^28–32^, which provides a global context to our regional analysis. Our approach is the first effort to quantify proportions of marine microbial endemism and cosmopolitanism in a way that is comparable to previous terrestrial studies^8^.

## Results

**Figure 2:**
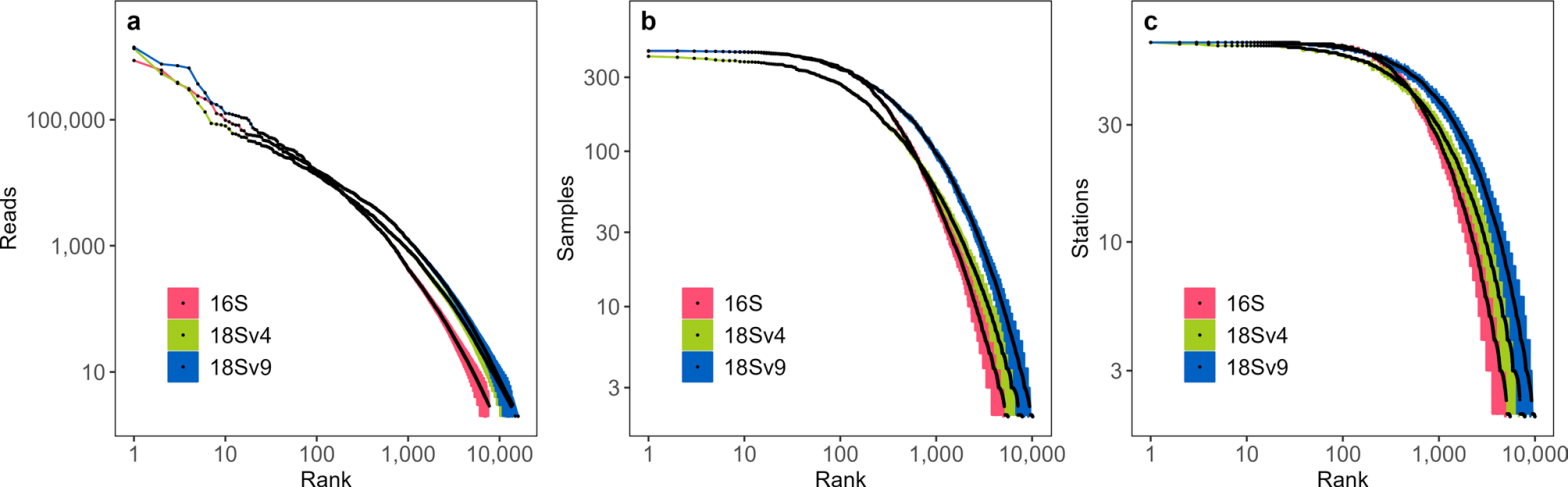
Rank curves for 16S, 18S-V4, and 18S-V9. (**a**) log_10_–log_10_ relationship between mean abundance (reads) and abundance rank. (**b**) log_10_–log_10_ relationship between mean occurrence (samples) and occurrence rank. (**c**) log_10_–log_10_ relationship between mean occurrence (stations) and occurrence rank. Color indicates either 16S (pink), 18S-V4 (green), or 18S-V9 (blue) ASVs. Shading around the means (points) show the upper (95%) and lower (5%) percentiles of either abundance or occurrence, calculated from the 1000-member rarefaction ensemble. ASVs that were observed with an average of < 1 read, sample, or station were removed from these figures for improved clarity.

The following results represent our analysis of a 1000-member ensemble (see “Methods” section) and reported values represent the mean value across all members or as a distribution of values across all members. Overall, we observed 13,558 distinct prokaryotic (16S) amplicon sequence variants (ASVs), as well as 21,095 18S-V4 ASVs and 25,815 18S-V9 ASVs from the respective eukaryotic datasets across the 1000-member ensembles in the Southern California Current region (SCC). The majority of ASVs across both prokaryotes and eukaryotes were rare in both total abundance and occurrence, where occurrence is defined as either occurring in a sample or station (Fig. 2). Over half of prokaryotic and eukaryotic ASVs had on average less than 10 reads, occurred in less than 3 samples, and occurred in less than 3 stations. ASVs that occurred in more samples were typically more abundant (Supplementary Fig. 1a). 50% of 16S, 57% of 18S-V4, and 53% 18S-V9 ASVs were found at most in only one sample.

To better understand ASVs that were found in one sample, referred to as singletons, we explored the distribution of reads for singletons as compared to all other ASVs (Supplementary Fig. 1b–d). The mean number of reads for singleton ASVs was 2.87 for 16S, 7.16 for 18S-V4, and 4.88 reads for 18S-V9. In contrast, the mean number of reads for all other ASVs was 1305 for 16S, 1011 for 18S-V4, and 1101 for 18S-V9. We used a Gaussian mixture model (mclust)^33^ to estimate the number of unique Gaussian distributions likely to make up the overall distribution for each dataset. While the best estimates ranged from 7 to 9 clusters, the largest increase in predictability (Bayesian information criterion, BIC) was from one to two clusters (Supplementary Fig. 2). The two clusters estimated from the Gaussian mixture models align well with the shift from singletons to taxa observed in more than one sample, indicating that some process (error or extreme rarity) is likely responsible for the unique dynamics of these singleton taxa (Supplementary Fig. 1b–d). Singletons were removed from the remaining analyses as it did not make sense to test the significance of habitat affinity for ASVs only observed once across the 445 samples. Thus, the following analyses were conducted across 6769 16S ASVs, 9072 18S-V4 ASVs, and 12,210 18S-V9 ASVs.

**Figure 3:**
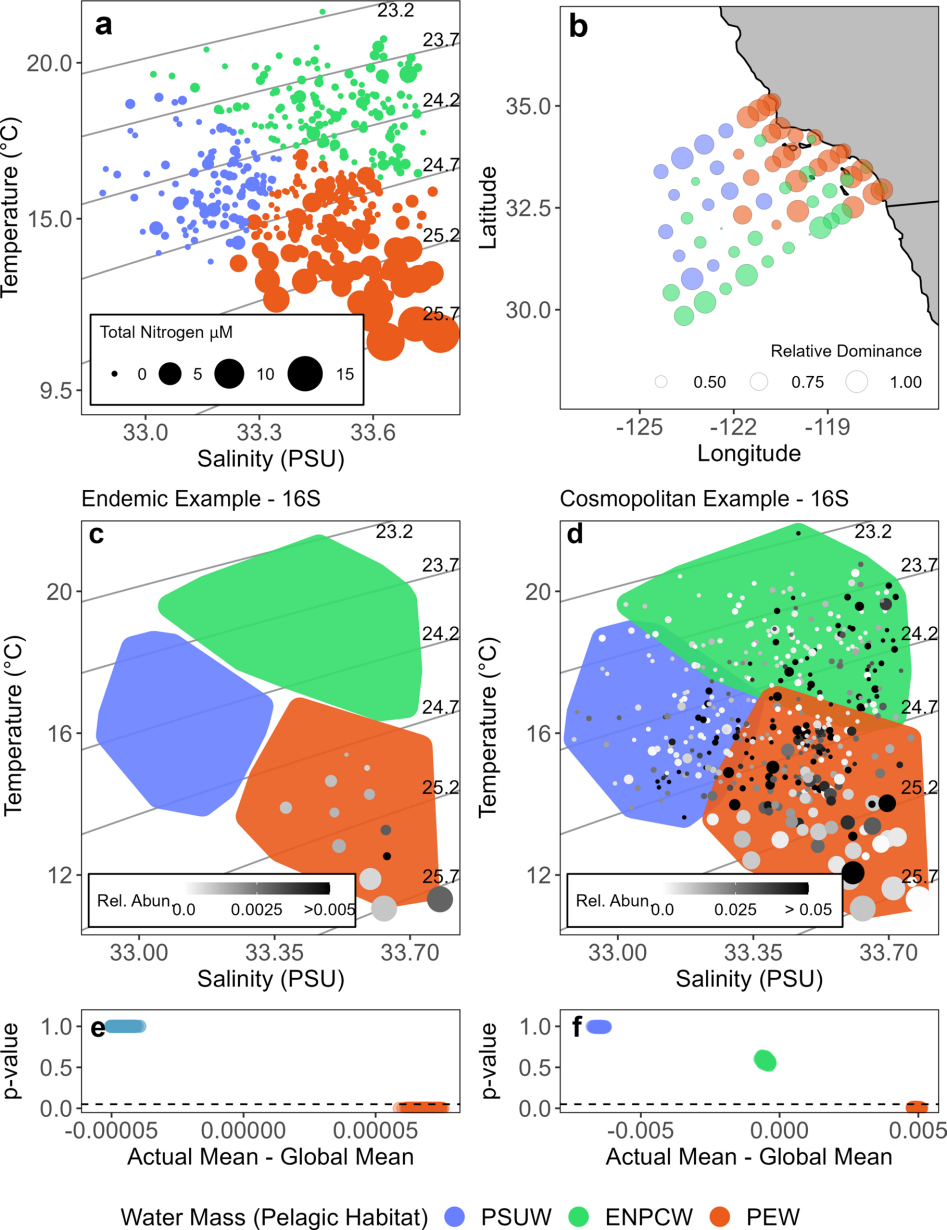
(**a**) Temperature, salinity, and dissolved inorganic nitrogen (NO_3_ + NH_3_) for the 445 samples used in this study. Axes show temperature (°C) and salinity (PSU). The size of the points represents NO_3_ + NH_3_ (µM) of each sample. Color of the points represents the identified SOM clusters which align with known water masses: Pacific Subarctic Upper Water (PSUW, blue), East North Pacific Central Water (ENPCW, green), and Pacific Equatorial Water (PEW, orange). Solid grey lines indicate isopycnals of constant seawater density (**c,d**). (**b**) Map showing the most dominant water mass per station (color of circles), where the size of the circles represents the frequency with which that water mass is observed at a given station. (**c,d**) Example temperature and salinity diagram showing the occurrence and relative abundance of an endemic (*Flavobacteriaceae* spp.) and cosmopolitan (*Synechococcus sp.* CC9902) ASV, respectively, across all 445 samples. The color of the points represents the relative abundance of the ASV per sample. Blue, green, and orange shaded regions show the boundary of each water mass/pelagic habitat. The size of the points represents NO_3_ + NH_3_ (µM) of each sample. (**e,f**) Example significance vs abundance diagrams for the endemic and cosmopolitan ASVs in (**c,d**), highlighting which pelagic habitats the ASVs had a significant affinity for (p-value < 0.05, dashed line). The x-axis shows the mean relative abundance within a pelagic habitat—the overall (across all samples) mean relative abundance for that ASV. The y-axis shows the p-value associated with each mean relative abundance per pelagic habitat (see “Methods” section for p-value calculation). A high value along the x-axis means that the abundance within a pelagic habitat is higher than the mean overall abundance for that taxon across all samples. Values below the dashed line on the y-axis represent ensemble members where the abundance was significantly greater in a pelagic habitat then the null (p-value < 0.05). Thus, in this example, the endemic ASV is significantly overabundant in the pelagic habitat it is observed in (PEW), while the cosmopolitan species, while found everywhere, is only significantly overabundant in the PEW.

Habitats, defined in the ocean by environmental properties such as temperature, salinity, and nutrients, move within the ocean. These physical and chemical water masses are the most comparable habitat descriptors to compare to terrestrial landscapes within the marine environment. Bograd et al.^24^ identified three main pelagic water masses, or habitats, in SCC region: the Pacific Subarctic Upper Water (PSUW; relatively fresh, cool, and nutrient-poor), the East North Pacific Central Water (ENPCW; warm, salty, and nutrient-poor), and the Pacific Equatorial Water (PEW; cool, salty, and nutrient-rich). Our SOM clustering approach separated samples into these three water masses using temperature, salinity, and dissolved nitrogen (NO_3_ + NH_3_) data (Fig. 3a,b). The SOM clusters matched the physical characteristics and spatial distributions of these three water masses described by Bograd et al.^24^.

ASVs were then categorized into three biogeographic classes based on their distribution across these pelagic habitats: endemic, cosmopolitan, and generalist. Endemic ASVs were defined as ASVs that were only present in one pelagic habitat across all 1000 rarefaction ensemble members (see Fig. 3c for an endemic example). Cosmopolitan ASVs were defined as ASVs that were present in at least one sample of all three pelagic habitats across all 1000 rarefaction ensemble members (see Fig. 3d for a cosmopolitan example). Generalist ASVs were defined as ASVs that only occur in two pelagic habitats or are sometimes seen in one or three pelagic habitats but not across all 1000 iterations. Generalist in this context therefore means the taxon is found in multiple but not all pelagic habitats.

**Figure 4:**
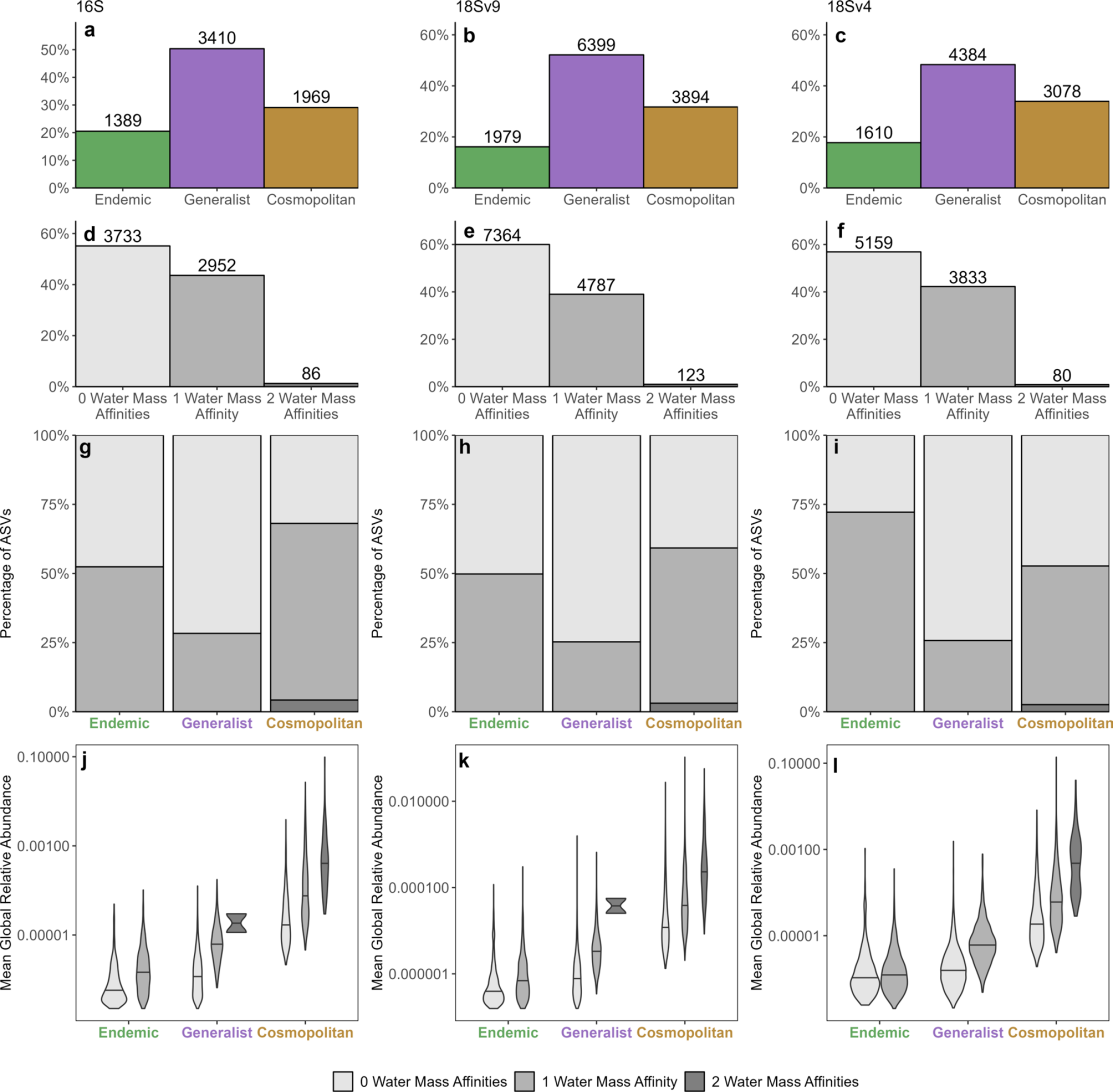
(**a–c**) Percentage of 16S, 18S-V9, and 18S-V4 ASVs respectively, in each biogeographic category (Endemic, Generalist, and Cosmopolitan). Values above each bar show the number of ASVs in each category. (**d–f**) Percentage of 16S, 18S-V9, and 18S-V4 ASVs respectively, in each affinity (zero habitat affinities, one habitat affinity, or two habitat affinities). Values above each bar show the number of ASVs in each biogeographic category. (**g–i**) Percentage of 16S, 18S-V9, and 18S-V4 ASVs respectively, in each habitat affinity level per biogeographic category. (**j–l**) Distributions of mean overall (across all samples) relative abundance across all 16S, 18S-V9, and 18S-V4 ASVs respectively. Distributions are separated per descriptive category and affinity level. Shades of gray in (**g–i**) correspond to the shades of gray for habitat affinities used in (**d–f**).

ASVs exhibited biogeographic patterns ranging from endemic to cosmopolitan. 50.4% of 16S, 48.3% of 18S-V4, and 52.1% of 18S-V9 ASVs were found in more than one pelagic habitat, but not in all three (i.e., “generalist” taxa; Fig. 4a–c). Cosmopolitanism was the next most common distribution with 29.1% of 16S ASVs, 33.9% of 18S-V4 ASVs, and 31.7% of 18S-V9 ASVs occurring in all three pelagic habitats. Endemic proportions were 20.5% for 16S ASVs, 17.7% for 18S-V4 ASVs, and 16.1% of 18S-V9 ASVs. Ubiquity, i.e., occurring in all samples, was very rare with only three 16S ASVs and two 18S-V9 ASVs out of 6,769 and 12,210 total ASVs, respectively, found in all samples. No 18S-V4 ASVs were found in all samples.

Following this biogeographic classification, we tested whether ASVs were significantly overabundant in pelagic habitats, hereafter referred to as habitat affinity. The mean relative abundance per ASV per pelagic habitat was compared against a null distribution to assess whether ASVs had a significant affinity towards any pelagic habitat (see “Methods” for details). In other words, is an ASV relatively more abundant than expected due to random chance in a given pelagic habitat? This test was used as a significance test for endemism (see Fig. 3e for an endemic example) and to explore overall habitat affinity for pelagic habitats across all ASVs (see Fig. 3f for a cosmopolitan example).

Taxa showed various levels of habitat affinity, from no affinity up to an affinity for two of the three habitats in this region (maximum). ASVs that occurred more or less evenly across all three habitats had no habitat affinity, hence a maximum habitat affinity of two. An ASV with no affinity for a particular pelagic habitat also meant that its distribution did not differ significantly from random. 55.1% of 16S ASVs, 56.9% of 18S-V4 ASVs, and 60.0% of 18S-V9 ASVs had no significant habitat affinity (Fig. 4d–f). The bulk of ASVs with no habitat affinities were generalists (Fig. 4g–i)—65.5% for prokaryotes (16S), and 63.1% (18S-V4) and 64.9% (18S-V9) for the two eukaryotic datasets. Generalist ASVs with no habitat affinities tended to have a similar rarity to endemics but appeared randomly distributed (Fig. 4j–l). Of the remaining ASVs with some level of habitat affinity, 43.6% (16S), 42.3% (18S-V4), and 39.0% (18S-V9) had an affinity for one pelagic habitat within the region. Only 86 16S ASVs, 80 18S-V4 ASVs, and 123 18S-V9 ASVs had an affinity for two pelagic habitats.

One question that arises is whether there are observed relationships between overall abundance and either biogeographic patterns or habitat affinity. For both prokaryotic and eukaryotic ASVs, there was a significant relationship (nested ANOVA, p < 0.001) between overall (across all samples) mean relative abundance and biogeographic patterns (Endemic, Generalist, Cosmopolitan). Endemic ASVs were the most rare and cosmopolitan ASVs were the most common (Fig. 4j–l). There was also a significant nested effect within each category (Endemic, Generalist, Cosmopolitan) wherein the overall mean relative abundance for both prokaryotes and eukaryotes was higher as ASVs went from no habitat affinities up to two habitat affinities within each category (Endemic, Generalist, Cosmopolitan, nested ANOVA, p < 0.001 for both). ASVs with higher overall relative abundance had affinities for more pelagic habitats. The opposite is also true: ASVs with no pelagic habitat affinity (that is, most ASVs), were the rarest.

**Figure 5:**
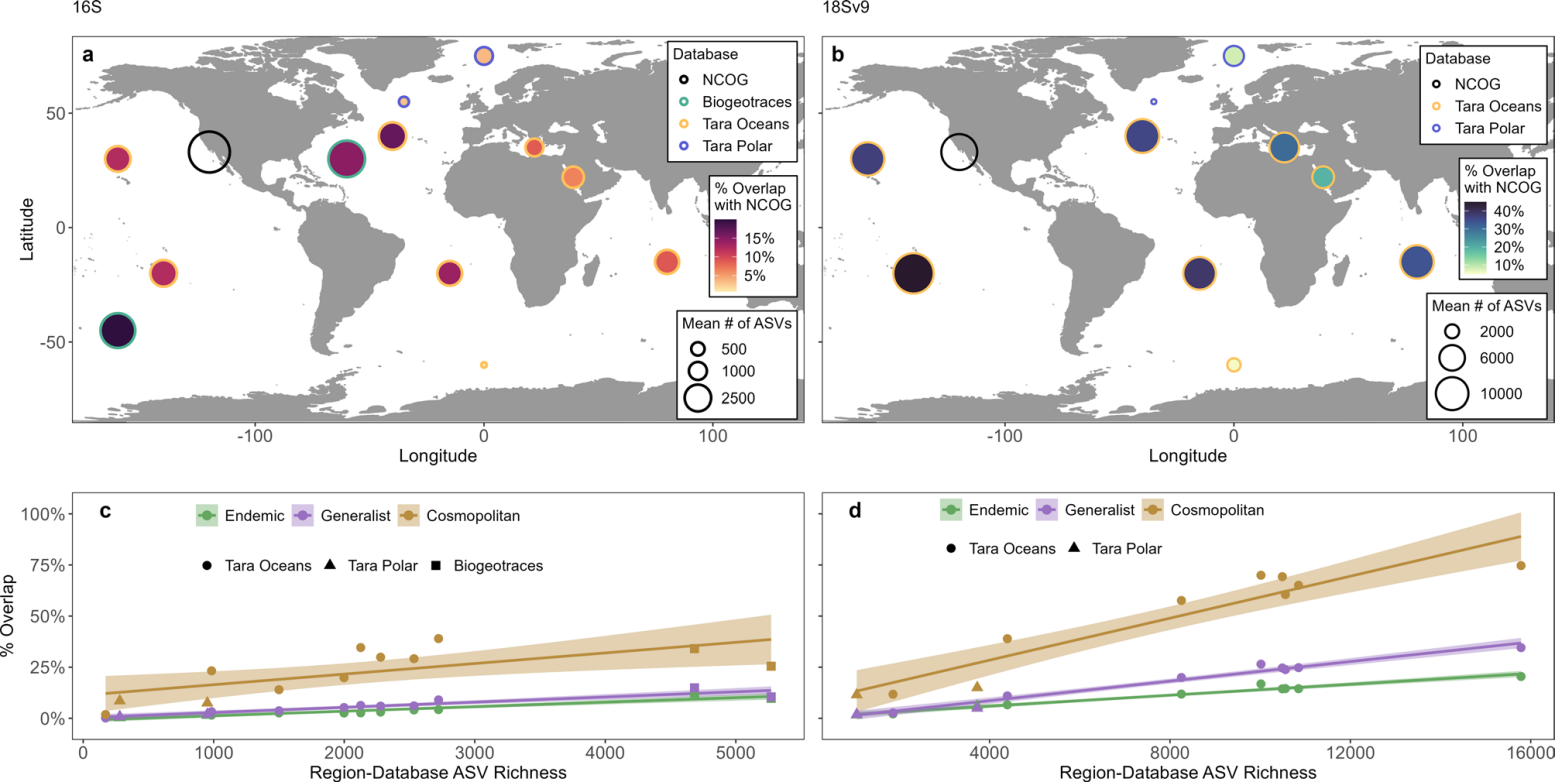
(**a,b**) Taxonomic overlap between NCOG and BioGEOTRACES, Tara Oceans, and Tara Polar for 16S (**a**) and Tara Oceans and Tara Polar for 18S-V9 (**b**). Size of circles indicates the mean number of ASVs identified per region per database. Edge color represents the database for the respective data (NCOG, BioGEOTRACES, Tara Ocean, or Tara Polar). Fill color represents the percentage of NCOG ASVs found in each respective region/dataset. (**c,d**) Relationship between regional richness (per database) and the % overlap between NCOG 16S ASVs and regional 16S and 18S-V9 ASVs. Each point represents a different region, as seen in (**a,b**). Colors represent the three biogeographic categories (Endemic, Generalist, Cosmopolitan). Linear fits between region-database richness and percent overlap were derived from the glm package in R.

Finally, we quantified the degree of taxonomic overlap between our NCOG data and global survey data (Tara Oceans, Tara Polar, and BioGEOTRACES) to place results from this region in a broader context. For this portion, only 16S and 18S-V9 data were used to align with the primers used in global datasets. Note that for Tara 16S samples, particle-associated bacteria may be absent as the result of their size-fractionation sampling approach. Hence, absolute values of overlap between NCOG 16S and Tara 16S may be underestimated. On average across rarefaction ensembles, 30.0% of prokaryotic ASVs (16S) and 16.8% of NCOG eukaryotic ASVs (18S-V9) were not found in any global samples (Tara and BioGEOTRACES combined). Overlap varied widely between ocean/sea basins, and below we broadly highlight general differences between these basins (for more detailed overlaps between specific basins/databases see Fig. 5). Taxonomic overlap among NCOG and Tara Ocean and BioGEOTRACES surveys was highest in the Pacific and Atlantic basins, 15.0% and 39.9% on average for prokaryotes and eukaryotes respectively (Fig. 5a,b). Intermediate levels of taxonomic overlap occurred between NCOG and the Indian Ocean, Red Sea, and Mediterranean Sea, 8.1% and 27.9% on average for prokaryotes and eukaryotes respectively. Lowest levels of overlap occurred between NCOG and the Southern Ocean, Arctic Ocean, and Tara Polar North Atlantic samples, 2.4% and 6.2% on average for prokaryotes and eukaryotes respectively (Fig. 5a,b). In general, we found that the degree of overlap between NCOG and global sampling regions aligned strongly with the total diversity found in each region, with higher regional diversity (# of ASVs) associated with a higher overlap (Fig. 5c,d).

We also explored the taxonomic overlap between ASVs in biogeographic categories (Endemic, Generalist, and Cosmopolitan) asking whether ASVs that were regionally cosmopolitan were more likely to occur globally than ASVs that represented fewer habitats (Generalists or Endemics). Eukaryotic cosmopolitan NCOG ASVs had the most striking relationship between regional diversity and overlap; low diversity regions contained few of the eukaryotic cosmopolitan NCOG ASVs while high diversity regions contained up to 74.7% of the NCOG eukaryotic cosmopolitans (Fig. 5d).

Overlap between NCOG and global samples varied among taxonomic groups and from region to region, as certain prokaryotic ASVs (Supplementary Fig. 3) and eukaryotic ASVs (Supplementary Fig. 4) found in NCOG were more or less likely to be found globally. Within prokaryotes, *Thermoplasmata* ASV richness was much higher across global regions relative to NCOG for all regions but the Southern Ocean. Within the Southern Ocean, the limited overlap was driven almost completely by the three most diverse prokaryotic groups (*Alphaproteobacteria*, *Gammaproteobacteria*, and *Bacteroidia*, Supplementary Fig. 3b). Within eukaryotes, taxonomic groups were more variable from region to region than prokaryotes. Eukaryotic groups that represented more of the overall habitat richness in their region relative to their dominance in NCOG included: *Choanoflagellatea*, *Prymnesiophyceae*, and *Filosa-Imbricatea*. The overlap between eukaryotic NCOG ASVs and eukaryotic Southern Ocean ASVs was far more diverse than for prokaryotic groups, even though the overlap (# of ASVs) between NCOG and the Southern Ocean was small (Supplementary Figs. 3b, 4b).

## Discussion

Overall, we found that the majority of ASVs identified within the Southern California Current Ecosystem (CCE) were rare in both occurrence and abundance (Fig. 2). Roughly half of the prokaryotic and eukaryotic diversity was represented by singletons ASVs that only occurred in one sample. While some singletons may represent real observations, we believe that a significant proportion of these singletons are the result of random errors along the sequencing and analysis pipeline^34,35^. Within the non-singleton ASVs, the majority had no habitat specificity (Fig. 4). These ASVs were relatively rare (Fig. 4j–l) and on average had less taxonomic overlap with global sampling compared to those ASVs with habitat preferences (Fig. 5). These results align with previous findings which have highlighted the abundance of rare species within the marine microbiome^36–38^.

There are many environmental and ecological mechanisms that may explain the occurrence of the marine microbial rare biosphere in the Southern CCE^39–42^, including, but not limited to, immigration via dispersal, temporal storage effects, habitat heterogeneity, evolution, and dormancy. Dispersal of marine microbes from nearby or remote habitats can sustain local populations, even at very low levels^43^. Temporal storage effects^44^ occur when populations persist beyond conditions where their growth is optimal and could be important in a dynamic region like the CCE. Habitat heterogeneity promoted by submesoscale features in the ocean may enhance overall regional diversity and support a community structure with a few locally-dominant taxa with a low background of rarer species mixing from adjacent patches of distinct ocean habitats^25,26^. Marine microbial population sizes are very large and generation times short, such that evolution via natural selection on standing genetic diversity and new mutations occurs on relatively short timescales^45,46^, and may support the rare microbial biosphere when new genotypes are introduced. Finally, many marine microbes exhibit dormant phases that may persist for long periods of time in suboptimal conditions and well outside their optimal habitats^47,48^. While we are unable in this context to identify the primary mechanisms supporting the rare microbial biosphere, it is an essential feature of the marine microbiome in the Southern CCE.

Generalist ASVs, observed in two of the three habitats within the region, were the most common biogeographic category. The majority of generalist ASVs had no habitat affinity, meaning that they were randomly distributed between the habitats they were observed in (Fig. 4). The observed pattern of generalists could be the result of many factors. Two of the three habitats (ENPCW and PSUW, Fig. 3) are more oligotrophic, thus, generalists that are present across these two habitats may represent species with wide niche tolerances across certain gradients (temperature, salinity) while maintaining a more selective niche across other gradients (nutrients). Dispersal and plasticity could also be responsible for the pattern found within generalists, where species appear randomly distributed across multiple habitats^43,49^. On average, generalists were more abundant than endemics but less abundant than cosmopolitan species. While generalists were the most common category found in the region, they are also the most unconstrained, as their distributions likely represent many different organisms, strategies, and processes within the pelagic environment.

Cosmopolitan ASVs were the second most common biogeographical category. Most cosmopolitans had an affinity for one pelagic habitat and were more abundant compared to endemics or generalists (Fig. 4). Large abundances combined with habitat preferences may indicate that mass effects (the diffusion of populations from areas of high to low density) may drive their cosmopolitan distributions at the regional scale^43^. Vigorous currents and cross-shore flux in this region^50,51^ likely facilitate horizontal dispersal of microbes across habitats to the extent that certain microbes are found in all regional pelagic habitats. These cosmopolitan taxa may also have relatively broad ecological niches^46,52^ or high phenotypic or trait plasticity^53,54^, such that they may retain positive net growth in all habitats. Other mechanisms, such as dormancy^47^ and predation reduction^55^, may also facilitate cosmopolitan distributions. ASVs that were regionally cosmopolitan in NCOG were far more likely to occur across the global regions sampled in the TARA databases (Fig. 5). Overlap between NCOG cosmopolitans and global datasets was greatest in regions of high diversity (Atlantic and Pacific). In contrast, regions of low total diversity had the lowest overlap with NCOG cosmopolitans and could correspond to either dispersal bottlenecks (Mediterranean and Red Sea) and or strong environmental gradients (Arctic and Southern Ocean) presenting strong enough barriers to the immigration of NCOG cosmopolitans. However, it is important to note that these results may not extend to particle associated microbes. Overall, these results suggest that cosmopolitan taxa are a common, but by no means dominant, component of the marine microbiome.

Endemic ASVs were the third most common biogeographic category and were evenly split between ASVs that appeared in only one habitat but had no significant affinity for that habitat, and ASVs that were both overabundant and endemic to one habitat. The exception being 18S-V4 endemic ASVs which tended to have a higher degree of habitat affinity relative to the other amplicons. This could be the result of 18S-V4 having a higher taxonomic resolution relative to 16S and 18S-V9. The levels of endemism in this system are drastically lower than in terrestrial systems where the endemic proportion of the community can be around 40% on a sample-by-sample basis and over 80% of regional richness^56^. Terrestrial systems likely have a higher prevalence of endemism when compared to marine systems due to multiple factors. Geographic barriers such as mountains and oceans restrict dispersal in terrestrial habitats and are far less common in pelagic systems^21^. Terrestrial habitats can also be spatially conserved on the relevant evolutionary timescales for species to become well adapted to particular locales^57^. In contrast, pelagic organisms deal with an ever-changing landscape and habitat displacement—leading to realized niches (niches as observed in nature) that are wider than their fundamental niches (preferred niche under controlled conditions)^58^.

In this study, we leveraged a unique, regional survey of the marine microbiome to quantify the prevalence of endemic, cosmopolitan, and generalist taxa and their habitat affinities. The marine microbiome in this region was characterized by a “rare biosphere” of many taxa with low abundance and occurrence. Many cosmopolitan taxa were distributed throughout the region across all pelagic habitats, and a smaller proportion of endemic taxa were found in only one pelagic habitat. Most taxa were found in more than one pelagic habitat, and among the generalist and cosmopolitan taxa that were found in multiple habitats, it was rare to show a habitat affinity for more than one habitat. Overall, this study supports recent findings that the majority of marine microbes are unlikely to be globally ubiquitous^14,59^. Rather, they show distinct biogeographies that are driven by the same core ecological processes of dispersal, selection, and drift, but perhaps at different scales compared to macroorganisms and terrestrial systems^60^.

## Methods

### Study location and sample collection

NOAA-CalCOFI Genomics Project (NCOG) data were collected within the Southern California Current (SCC) region, a productive eastern boundary current ecosystem. The data consists of 445 near-surface (nominally 10m depth) samples collected quarterly from 2014 to 2020. Cardinal stations on CalCOFI lines 80 (stations 55.0, 70.0, 80.0, 100.0), 81.8 (station 46.9), and 90 (stations 37.0, 53.0, 70.0, 90.0, 120.0) were sampled every cruise. Primary productivity stations, which measure ^14^C primary production at approximately local noon were also sampled. The locations of primary productivity stations vary from cruise to cruise depending on where the ship is located each day at approximately local noon.

Both molecular and environmental data were collected via a CTD rosette. Temperature and salinity were measured with the Seabird 911 CTD. Salinity measurements were compared to bottle samples that were measured with a Guildline Portasal Salinometer model 8410A. Nutrients (NO_3_ and NH_3_) were measured with a QuAAtro continuous flow autoanalyzer (SEAL analytical). For a comprehensive description of collection and processing methods related to the NCOG database, see James et al.^27^.

### DNA collection and extraction

2 L or more of seawater (4.4 L on average) was filtered through a 0.22 µm Sterivex-GP filter unit (MilliporeSigma, Burlignton, MA, USA). For 7 out of the 445 samples, less than 2 L of seawater was filtered, primarily due to the high biomass in these samples. Samples were immediately sealed with a sterile luer-lock plug and hematocrit sealant, wrapped in aluminum foil, and flash frozen in liquid nitrogen. DNA was extracted with the NucleoMag Plant Kit for DNA purification (Macherey–Nagel, Düren, Germany) on an epMotion 5075TMX (Eppendorf, Hamburg, Germany) as described here: 10.17504/protocols.io.bc2hiyb6. DNA was assessed on a 1.8% agarose gel after extraction.

### Amplicon sequencing and analysis

Amplicon sequence variant (ASV) libraries used in this analysis targeted the V4–V5 region of the 16S rRNA gene for prokaryotes and the V4 and V9 regions of the 18S rRNA gene for eukaryotes. For both the 18S-V4 and 18S-V9 only 5% of eukaryotic ASVs were prokaryotes and other microbial eukaryotes, and therefore the bulk of our results and discussion focus on microbes, which includes archaea, bacteria, and eukaryotic protists.

DNA was amplified with a one-step PCR using a TruFi DNA Polymerase PCR kit (Azura, Raynham, MA, USA). For 16S, the 515 F-Y/926R primers from Parada et al. 2016 were used^61^. For 18S-V4, V4F/V4RB primers from Berdjeb et al.^62^ were used. For 18S-V9, 1389F/1510R primers from Amaral-Zettler et al. 2009^63^ were used. Each reaction included an initial denaturing step at 95 °C for 1 min followed by 30 cycles of 95 °C for 15 s, 56 °C for 15 s, and 72 °C for 30 s. Custom mock communities were included in all sequencing runs^61^. Extraction blanks and negative controls were also included in PCR and sequencing and produced very little DNA and resulting reads. Next, 2.5 µL of each PCR reaction was then ran on a 1.8% agarose gel to confirm amplification. Following this, PCR products were purified using Beckman Coulter AMPure XP beads as dictated by the standard 1× PCR clean-up protocol. PCR quantification was performed in duplicate using Invitrogen Quant-iT PicoGreen dsDNA Assay kits. Samples were pooled to 10 ng of purified PCR product before cleaning and concentrating the final library with 0.8× AMPure XP bead purification. Pools were evaluated on an Agilent 2200 TapeStation and quantified with Qubit HS dsDNA. Pools were sequenced at the University of California, Davis Sequencing core on a single Illumina MiSeq lane (2 × 300 bp for 16S and 18S-V4, and 1 × 150 bp for 18S-V9) with a 15% PhiX spike-in. For the 2014–2016 data, a V9 pool was sequenced on an Illumina NextSeq (2 × 150 bp).

Following sequencing, amplicons were analyzed with QIIME2 v2019.10^64^. Demultiplexed paired-end reads were tripped with cutadapt^65^ to remove adapter and primer sequences with the error rate option set to 0.1 and overlap option set to 3. Trimmed reads were denoised with DADA2 to produce amplicon sequence variants (ASVs) with the “pooled” chimera removal option^66^. Pools were analyzed individually to account for varying error profiles as the result of each run. Taxonomic annotation of ASVs was conducted with the q2-feature-classifier classify-sklearn naïve-bayes classifier^67,68^ against SILVA (Release 138)^69^ for 16S amplicons or PR^2^ v4.13.0^70^ for 18S-V4/V9 amplicons.

For protocols visit: https://www.protocols.io/view/amplicon-library-preparation-bmuck6sw.

### Rarefaction of amplicon data

Samples had a wide variety of library sizes. We rarefied our data to a consistent level of 20,024 reads after the removal of chloroplast and mitochondrial sequences for 16S, 21,933 reads for 18S-V4 and 30,347 reads for 18S-V9 for each respective eukaryotic dataset, to remove the effect of sequencing noise on our results. Repeated rarefaction steps were used to maintain novel but rare taxa, which is particularly important as we aimed to identify the prevalence of endemism, cosmopolitanism, and habitat specificity within the region^35^. We repeated the rarefaction step 1000 times and ran our analyses on each of these rarefied microbiome tables. Results for all figures represented the mean values across the entire 1000-member ensemble, with standard deviations highlighted where appropriate. In doing so, we were able to statistically assess the occurrence and habitat specificity of rare taxa that might not be observed through a single rarefaction step. This approach was used rather than other normalization approaches such as centered log ratio as the goal of the study was to explore the occurrence of organisms across space and time rather than changes between various taxa.

### Self-organizing maps

We used SOMs to differentiate marine habitats based upon environmental conditions. SOMs are a machine learning approach capable of reducing high dimensional data into a two-dimensional map^71^ and have been used to identify ‘seascapes’ along the Western Antarctic Peninsula^23^. We followed a similar procedure using three parameters: temperature, salinity, and dissolved nitrogen (NO_3_ + NH_3_) to construct our SOM. Once the SOM was generated, we used hierarchical clustering to cluster the SOM into three seascapes. The seascapes identified with our SOM aligned with the three major water masses that comprise surface waters in the SCC^24^. The spatial distribution of three core ocean habitats varied through time, reflecting the dynamic nature of the marine environment. We use the terms pelagic habitat, water mass, and seascape equivalently.

### Null model for pelagic habitat affinity

We used a null model to assess whether ASVs were more abundant within any given pelagic habitat, which we call habitat affinity. Null models were generated for each ASV. Below, we outline the processes for identifying habitat affinity for an individual taxon within each pelagic habitat for one ensemble member. This process was applied across all taxa and across all 1000 ensemble members.

We calculated the mean relative abundance of a taxon in each pelagic habitat using data across all cruises (${\overline{\mu }}_{ENPCW}, {\overline{\mu }}_{PSUW}, {\overline{\mu }}_{PEW}$). Then, the relative abundances of a taxon were reshuffled across all samples (with replacement) 1000 times. From these 1000 surrogate abundance distributions we calculated 1000 null mean relative abundances per pelagic habitat. P-values were calculated for actual mean relative abundances compared to their respective null distributions with the following equation:

1 $$\begin{array}{c}p= \frac{number\, of \,surrogate\, means>actual \,mean}{total\, number\, of \,surrogate \,means}.\end{array}$$

To assess the overall significance of pelagic habitat affinity within a taxon across all rarefaction ensemble members we calculated the mean p-value per pelagic habitat per taxa across all 1000 ensemble members.

### Global samples

In this study, we explored the overlap (shared occurrence) between NCOG, Tara Oceans, Tara Polar, and BioGEOTRACES ASVs. For both 16S and 18S-V9 TARA samples, we selected samples that were collected by filtering seawater via a peristaltic pump (excluding net samples) to keep the sampling as similar to our collection process for NCOG. For 16S, we looked at the size-fractioned filter from 0.22 to 1.6 or 3.0 µm (upper range varied across samples). For 18S-V9, this included multiple size fractions from 0.22 to 200 µm. Since NCOG does not use the size-fractionated methodology, this was the closest representation we could achieve to align TARA data to our own. BioGEOTRACES data was collected in a similar manner to NCOG.

## Supplementary Information

Supplementary Figures.

## Data Availability

The 16 S rDNA raw reads have been deposited at NCBI under Bioproject IDs PRJNA555783, PRJNA665326 and PRJNA804265 and Biosample accession nos. SAMN25705811-SAMN25706151, SAMN16250568-SAMN16251083, and SAMN25756929-SAMN25757078 for the 2014–2016, 2017–2019, and 2020 periods respectively. The 18 S rDNA raw reads have been deposited at NCBI under Bioproject IDs PRJNA555783, PRJNA665326, and PRJNA804265 and Biosample accession nos. SAMN25710021-SAMN25710361, SAMN16251281-SAMN16251796, and SAMN25757352-SAMN25757501 for the 2014–2016, 2017–2019, and 2020 periods respectively. Tara Oceans and Tara Polar 18S-V9 sequences can be found at the European Nucleotide Archive under the project accession IDs PRJEB6610 and PRJEB9737. Tara Oceans and Tara Polar 16S sequences can be found at the European Nucleotide Archive under the project accession IDs PRJEB4357, PRJEB36282, PRJEB36283, PRJEB36284, and PRJEB36285. BioGEOTRACES sequences can be found at NIH under the Bioproject ID PRJNA659851.

## References

[1] Field, C. B., Behrenfeld, M. J., Randerson, J. T. & Falkowski, P. Primary production of the biosphere: Integrating terrestrial and oceanic components. *Science* **281**, 237–240 (1998). 9657713 10.1126/science.281.5374.237

[2] Falkowski, P. G., Fenchel, T. & Delong, E. F. The microbial engines that drive earth’s biogeochemical cycles. *Microbe* **320**, 1 (2008). 10.1126/science.1153213 18497287

[3] Not, F. *et al.* Diversity and ecology of eukaryotic marine phytoplankton. *Adv. Bot. Res.* **64**, 1–53 (2012).

[4] Barton, A. D. *et al.* The biogeography of marine plankton traits. *Ecol. Lett.* **16**, 522–534 (2013). 23360597 10.1111/ele.12063

[5] Fuhrman, J. A. Microbial community structure and its functional implications. *Nature* **459**, 193–199 (2009). 19444205 10.1038/nature08058

[6] Gaston, K. J. Global patterns in biodiversity. *Nature* **405**, 1 (2000). 10.1038/35012228 10821282

[7] Fenchel, T. & Finlay, B. J. The ubiquity of small species: Patterns of local and global diversity. *Bioscience* **54**, 777–784 (2004).

[8] Kier, G. *et al.* A global assessment of endemism and species richness across island and mainland regions. *Proc. Natl. Acad. Sci. U.S.A.* **106**, 9322–9327 (2009). 19470638 10.1073/pnas.0810306106 PMC2685248

[9] Finlay, B. J. Global dispersal of free-living microbial eukaryote species. *Science* **296**, 1061 (2002). 12004115 10.1126/science.1070710

[10] Gibbons, S. M. *et al.* Evidence for a persistent microbial seed bank throughout the global ocean. *Proc. Natl. Acad. Sci.* **110**, 4651–4655 (2013). 23487761 10.1073/pnas.1217767110 PMC3607043

[11] Shurin, J. B., Cottenie, K. & Hillebrand, H. Spatial autocorrelation and dispersal limitation in freshwater organisms. *Oecologia* **159**, 1 (2009). 18941791 10.1007/s00442-008-1174-z

[12] Litchman, E. Invisible invaders: Non-pathogenic invasive microbes in aquatic and terrestrial ecosystems. *Ecol. Lett.* **13**, 1560 (2010). 21054733 10.1111/j.1461-0248.2010.01544.x

[13] Villarino, E. *et al.* Large-scale ocean connectivity and planktonic body size. *Nat. Commun.* **9**, 1 (2018). 29321528 10.1038/s41467-017-02535-8 PMC5762663

[14] Ward, B. A., Cael, B. B., Collins, S. & Robert Young, C. Selective constraints on global plankton dispersal. *Proc. Natl. Acad. Sci. U.S.A.* **118**, 1 (2021). 10.1073/pnas.2007388118 PMC7958371 33649201

[15] Morris, R. M. *et al.* SAR11 clade dominates ocean surface bacterioplankton communities. *Nature* **420**, 1 (2002). 10.1038/nature01240 12490947

[16] Brown, M. V. *et al.* Global biogeography of SAR11 marine bacteria. *Mol. Syst. Biol.* **8**, 595 (2012). 22806143 10.1038/msb.2012.28 PMC3421443

[17] Martiny, J. B. H. *et al.* Microbial biogeography: Putting microorganisms on the map. *Nat. Rev. Microbiol.* **4**, 102–112 (2006). 16415926 10.1038/nrmicro1341

[18] Malviya, S. *et al.* Insights into global diatom distribution and diversity in the world’s ocean. *Proc. Natl. Acad. Sci.* **113**, E1516–E1525 (2016). 26929361 10.1073/pnas.1509523113 PMC4801293

[19] Gimmler, A., Korn, R., De Vargas, C., Audic, S. & Stoeck, T. The Tara Oceans voyage reveals global diversity and distribution patterns of marine planktonic ciliates. *Sci. Rep.* **6**, 1–13 (2016). 27633177 10.1038/srep33555 PMC5025661

[20] Canals, O., Obiol, A., Muhovic, I., Vaqué, D. & Massana, R. Ciliate diversity and distribution across horizontal and vertical scales in the open ocean. *Mol. Ecol.* **29**, 2824–2839 (2020). 32618376 10.1111/mec.15528

[21] Costello, M. J. *et al.* Marine biogeographic realms and species endemicity. *Nat. Commun.* **8**, 1–9 (2017). 29051522 10.1038/s41467-017-01121-2 PMC5648874

[22] Kavanaugh, M. T. *et al.* Seascapes as a new vernacular for pelagic ocean monitoring, management and conservation. *ICES J. Mar. Sci.* **73**, 1839–1850 (2016).

[23] Bowman, J. S., Kavanaugh, M. T., Doney, S. C. & Ducklow, H. W. Recurrent seascape units identify key ecological processes along the western Antarctic Peninsula. *Glob. Change Biol.* **24**, 3065–3078 (2018). 10.1111/gcb.14161 29635875

[24] Bograd, S. J., Schroeder, I. D. & Jacox, M. G. A water mass history of the Southern California current system. *Geophys. Res. Lett.* **46**, 6690–6698 (2019).

[25] Lévy, M., Franks, P. J. S. & Smith, K. S. The role of submesoscale currents in structuring marine ecosystems. *Nat. Commun.* **9**, 4758 (2018). 30420651 10.1038/s41467-018-07059-3 PMC6232172

[26] Martín, P. V., Buček, A., Bourguignon, T. & Pigolotti, S. Ocean currents promote rare species diversity in protists. *Sci. Adv.* **6**, 1 (2020). 10.1126/sciadv.aaz9037 PMC7439499 32832617

[27] James, C. C. *et al.* Influence of nutrient supply on plankton microbiome biodiversity and distribution in a coastal upwelling region. *Nat. Commun.* **13**, 1–13 (2022). 35508497 10.1038/s41467-022-30139-4 PMC9068609

[28] de Vargas, C. *et al.* Eukaryotic plankton diversity in the sunlit ocean. *Science* **348**, 1261605 (2015). 25999516 10.1126/science.1261605

[29] Sunagawa, S. *et al.* Structure and function of the global ocean microbiome. *Science* **348**, 1–10 (2015). 10.1126/science.1261359 25999513

[30] Carradec, Q. *et al.* A global ocean atlas of eukaryotic genes. *Nat. Commun.* **9**, 1 (2018). 29371626 10.1038/s41467-017-02342-1 PMC5785536

[31] Ibarbalz, F. M. *et al.* Pan-Arctic plankton community structure and its global connectivity. *Elementa* **11**, 1 (2023).

[32] McNichol, J., Berube, P. M., Biller, S. J. & Fuhrman, J. A. Evaluating and improving small subunit rRNA PCR primer coverage for bacteria, archaea, and eukaryotes using metagenomes from global ocean surveys. *mSystems* **6**, 1 (2021). 10.1128/mSystems.00565-21 PMC8269242 34060911

[33] Scrucca, L., Fop, M., Murphy, T. B. & Raftery, A. E. Mclust 5: Clustering, classification and density estimation using Gaussian finite mixture models. *R J.* **8**, 289–317 (2016). 27818791 PMC5096736

[34] Berry, D. & Widder, S. Deciphering microbial interactions and detecting keystone species with co-occurrence networks. *Front. Microbiol.* **5**, 219 (2014). 24904535 10.3389/fmicb.2014.00219 PMC4033041

[35] Cameron, E. S., Schmidt, P. J., Tremblay, B. J. M., Emelko, M. B. & Müller, K. M. Enhancing diversity analysis by repeatedly rarefying next generation sequencing data describing microbial communities. *Sci. Rep.* **11**, 1–13 (2021). 34785722 10.1038/s41598-021-01636-1 PMC8595385

[36] Logares, R. *et al.* Patterns of rare and abundant marine microbial eukaryotes. *Curr. Biol.* **24**, 813–821 (2014). 24704080 10.1016/j.cub.2014.02.050

[37] Bachy, C. & Worden, A. Z. Microbial ecology: Finding structure in the rare biosphere. *Curr. Biol.* **24**, R315–R317 (2014). 24735853 10.1016/j.cub.2014.03.029

[38] Ser-Giacomi, E. *et al.* Ubiquitous abundance distribution of non-dominant plankton across the global ocean. *Nat. Ecol. Evol.* **2**, 1243–1249 (2018). 29915345 10.1038/s41559-018-0587-2

[39] Sogin, M. L. *et al.* Microbial diversity in the deep sea and the underexplored ‘rare biosphere’. *Proc. Natl. Acad. Sci. U.S.A.* **103**, 12115–12120 (2006). 16880384 10.1073/pnas.0605127103 PMC1524930

[40] Pedrós-Alió, C. Marine microbial diversity: Can it be determined? *Trends Microbiol.* **14**, 257–263 (2006). 16679014 10.1016/j.tim.2006.04.007

[41] Lynch, M. D. J. & Neufeld, J. D. Ecology and exploration of the rare biosphere. *Nat. Rev. Microbiol.* **13**, 217–229 (2015). 25730701 10.1038/nrmicro3400

[42] Pascoal, F., Costa, R. & Magalhães, C. The microbial rare biosphere: Current concepts, methods and ecological principles. *FEMS Microbiol. Ecol.* **97**, 227 (2021). 10.1093/femsec/fiaa227 33175111

[43] Leibold, M. A. *et al.* The metacommunity concept: A framework for multi-scale community ecology. *Ecol. Lett.* **7**, 601–613 (2004).

[44] Cáceres, C. E. Temporal variation, dormancy, and coexistence: A field test of the storage effect. *Proc. Natl. Acad. Sci. U.S.A.* **94**, 1 (1997). 11038565 10.1073/pnas.94.17.9171 PMC23092

[45] Lohbeck, K. T., Riebesell, U. & Reusch, T. B. H. Adaptive evolution of a key phytoplankton species to ocean acidification. *Nat. Geosci.* **5**, 346–351 (2012).

[46] Irwin, A. J., Finkel, Z. V., Müller-Karger, F. E. & Ghinaglia, L. T. Phytoplankton adapt to changing ocean environments. *Proc. Natl. Acad. Sci. U.S.A.* **112**, 5762–5766 (2015). 25902497 10.1073/pnas.1414752112 PMC4426419

[47] Jones, S. E. & Lennon, J. T. Dormancy contributes to the maintenance of microbial diversity. *Proc. Natl. Acad. Sci. U.S.A.* **107**, 5881–5886 (2010). 20231463 10.1073/pnas.0912765107 PMC2851880

[48] Lennon, J. T. & Jones, S. E. Microbial seed banks: The ecological and evolutionary implications of dormancy. *Nat. Rev. Microbiol.* **9**, 119–130 (2011). 21233850 10.1038/nrmicro2504

[49] Litchman, E., Edwards, K. F., Klausmeier, C. A. & Thomas, M. K. Phytoplankton niches, traits and eco-evolutionary responses to global environmental change. *Mar. Ecol. Prog. Ser.* **470**, 235–248 (2012).

[50] Chabert, P., d’Ovidio, F., Echevin, V., Stukel, M. R. & Ohman, M. D. Cross-shore flow and implications for carbon export in the california current ecosystem: A Lagrangian analysis. *J. Geophys. Res. Ocean* **126**, e2020JC016611 (2021).

[51] Giddings, A., Franks, P. J. S. & Baumann-Pickering, S. Monthly to decadal variability of mesoscale stirring in the california current system: Links to upwelling, climate forcing, and chlorophyll transport. *J. Geophys. Res. Ocean* **127**, e2021JC018180 (2022).

[52] Thomas, M. K., Kremer, C. T., Klausmeier, C. A. & Litchman, E. A global pattern of thermal adaptation in marine phytoplankton. *Science* **338**, 1085–1088 (2012). 23112294 10.1126/science.1224836

[53] Hattich, G. S. I. *et al.* Inter- and intraspecific phenotypic plasticity of three phytoplankton species in response to ocean acidification. *Biol. Lett.* **13**, 1 (2017). 10.1098/rsbl.2016.0774 PMC5326507 28148833

[54] Kwon, E. Y. *et al.* Nutrient uptake plasticity in phytoplankton sustains future ocean net primary production. *Sci. Adv.* **8**, 1 (2022). 10.1126/sciadv.add2475 PMC9770953 36542698

[55] Rigby, K. & Selander, E. Predatory cues drive colony size reduction in marine diatoms. *Ecol. Evol.* **11**, 11020–11027 (2021). 34429899 10.1002/ece3.7890 PMC8366847

[56] Talbot, J. M. *et al.* Endemism and functional convergence across the North American soil mycobiome. *Proc. Natl. Acad. Sci. U.S.A.* **111**, 6341–6346 (2014). 24733885 10.1073/pnas.1402584111 PMC4035912

[57] Rosauer, D. F. & Jetz, W. Phylogenetic endemism in terrestrial mammals. *Glob. Ecol. Biogeogr.* **24**, 168–179 (2015).

[58] Smith, A. N. *et al.* Comparing Prochlorococcus temperature niches in the lab and across ocean basins. *Limnol. Oceanogr.* **66**, 2632–2647 (2021).

[59] van der Gast, C. J. Microbial biogeography: The end of the ubiquitous dispersal hypothesis? *Environ. Microbiol.* **17**, 544–546 (2015). 25521363 10.1111/1462-2920.12635

[60] Vellend, M. Conceptual synthesis in community ecology. *Q. Rev. Biol.* **85**, 183–206 (2010). 20565040 10.1086/652373

[61] Parada, A. E., Needham, D. M. & Fuhrman, J. A. Every base matters: Assessing small subunit rRNA primers for marine microbiomes with mock communities, time series and global field samples. *Environ. Microbiol.* **18**, 1 (2016). 26271760 10.1111/1462-2920.13023

[62] Berdjeb, L., Parada, A., Needham, D. M. & Fuhrman, J. A. Short-term dynamics and interactions of marine protist communities during the spring–summer transition. *ISME J.* **12**, 1907–1917 (2018). 29599520 10.1038/s41396-018-0097-x PMC6052004

[63] Amaral-Zettler, L. A., McCliment, E. A., Ducklow, H. W. & Huse, S. M. A method for studying protistan diversity using massively parallel sequencing of V9 hypervariable regions of small-subunit ribosomal RNA Genes. *PLoS ONE* **4**, 1 (2009). 10.1371/journal.pone.0006372 PMC2711349 19633714

[64] Bolyen, E. *et al.* Reproducible, interactive, scalable and extensible microbiome data science using QIIME 2. *Nat. Biotechnol.* **37**, 852–857 (2019). 31341288 10.1038/s41587-019-0209-9 PMC7015180

[65] Martin, M. Cutadapt removes adapter sequences from high-throughput sequencing reads. *EMBnet J.* **17**, 1 (2011).

[66] Callahan, B. J. *et al.* DADA2: High-resolution sample inference from Illumina amplicon data. *Nat. Methods* **13**, 1 (2016). 10.1038/nmeth.3869 PMC4927377 27214047

[67] Bokulich, N. A. *et al.* Optimizing taxonomic classification of marker-gene amplicon sequences with QIIME 2’s q2-feature-classifier plugin. *Microbiome* **6**, 1 (2018). 29773078 10.1186/s40168-018-0470-z PMC5956843

[68] Pedregosa, F. *et al.* Scikit-learn: Machine learning in python. *J. Mach. Learn. Res.* **12**, 1 (2011).

[69] Pruesse, E. *et al.* SILVA: A comprehensive online resource for quality checked and aligned ribosomal RNA sequence data compatible with ARB. *Nucleic Acids Res.* **35**, 1 (2007). 17947321 10.1093/nar/gkm864 PMC2175337

[70] Guillou, L. *et al.* The Protist Ribosomal Reference database (PR2): A catalog of unicellular eukaryote small sub-unit rRNA sequences with curated taxonomy. *Nucleic Acids Res.* **41**, D597 (2013). 23193267 10.1093/nar/gks1160 PMC3531120

[71] Kohonen, T. Exploration of very large databases by self-organizing maps. *IEEE Int. Conf. Neural Netw. Conf. Proc.* **1**, 1 (1997).

