# Supplementary material for: Endemic, cosmopolitan, and generalist taxa and their habitat affinities within a coastal marine microbiome
Source: Sci Rep. 2024 Sep 28;14:22408. doi: 10.1038/s41598-024-69991-3 (PMC11437011; doi:10.1038/s41598-024-69991-3)
Supplement: Supplementary file 1 — Supplementary Figures. [file 41598_2024_69991_MOESM1_ESM.docx]

**
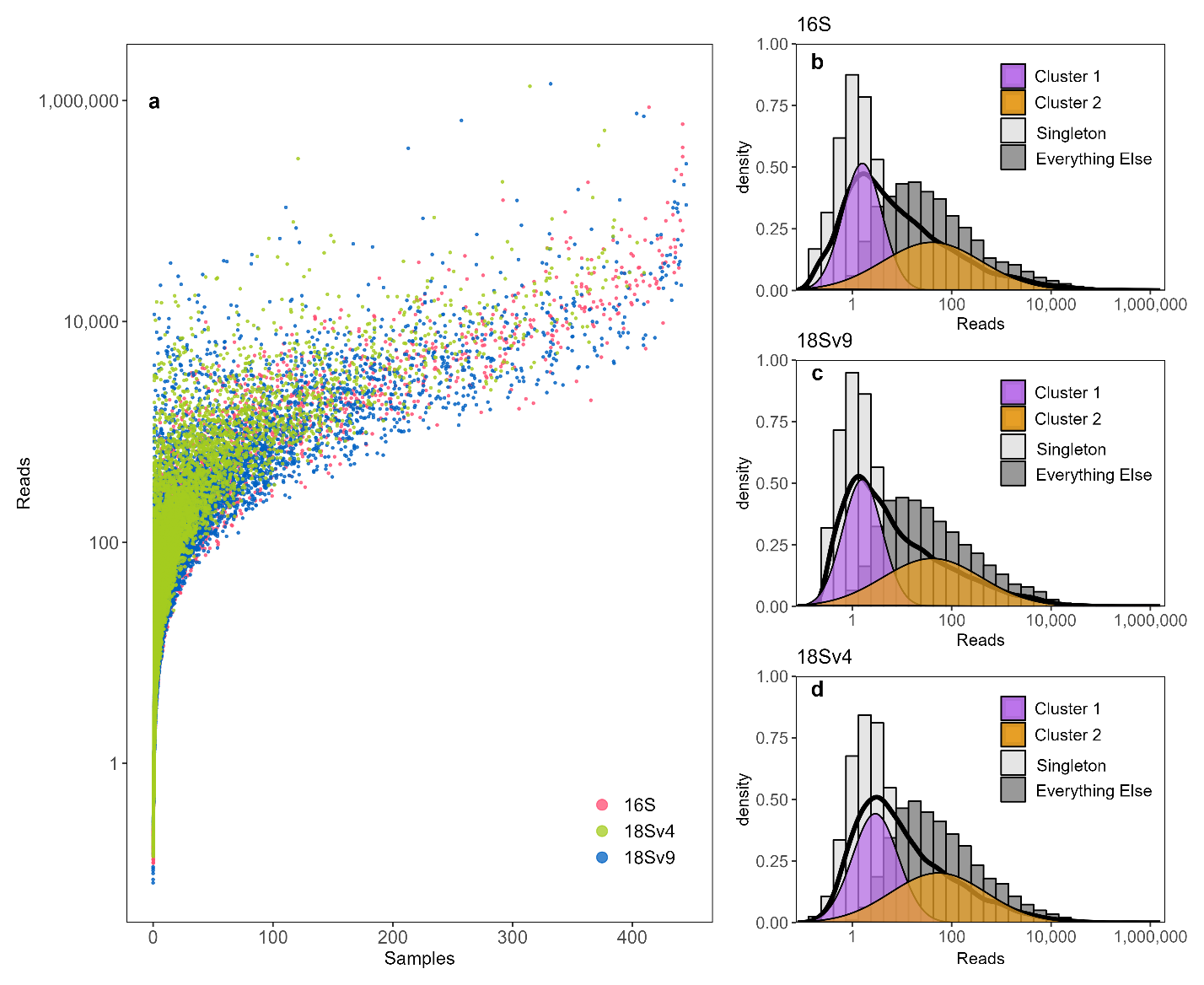
**

**Supplementary Figure 1: a**, Number of samples versus log_10_ number of reads, or abundance, for 16S, 18S-V4, and 18S-V9. Color indicates either 16S (pink), 18S-V4 (green), or 18S-V9 (blue) ASVs. **b**, Histogram of mean total reads (log_10_) per ASV split between Singletons (ASVs only seen in one sample, light grey) and all other ASVs (dark grey) for 16S. The density outline (thick black line) shows the distribution of all 16S reads while filled purple and orange distributions represent clusters 1 and 2 within a Gaussian mixed model. **c.** Histogram of mean total reads (log_10_) per ASV as shown in panel b for 18S-V9 ASVs. d**.** Histogram of mean total reads (log_10_) per ASV as shown in panel b for 18S-V4 ASVs.


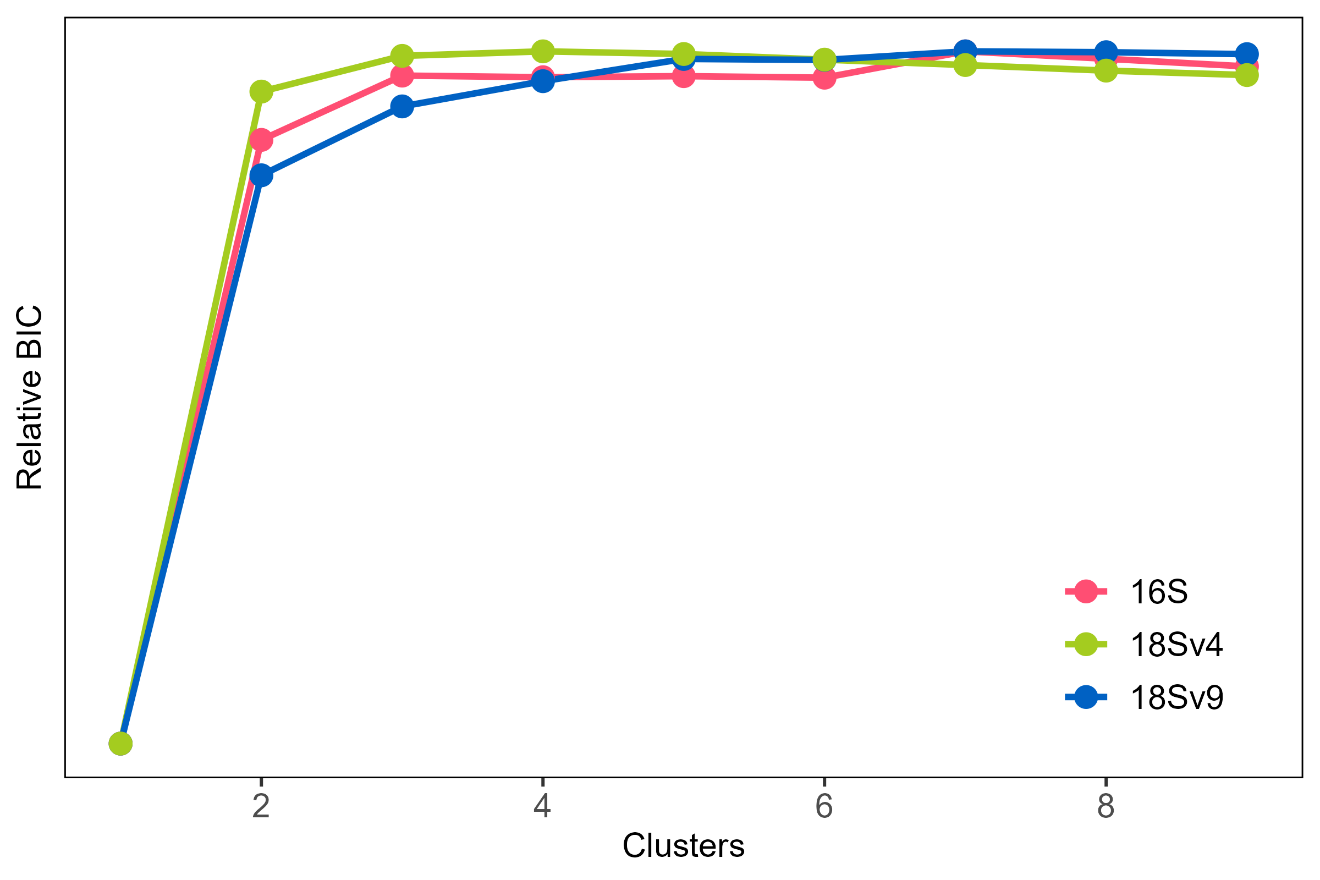


**Supplementary Figure 2:** Relative BIC values for the possible number of clusters derived from a Gaussian mixed model (mclust, Scrucca *et al.* n.d. ). Colors indicate 16S (red), 18S-V4 (green), and 18S-V9 (blue).


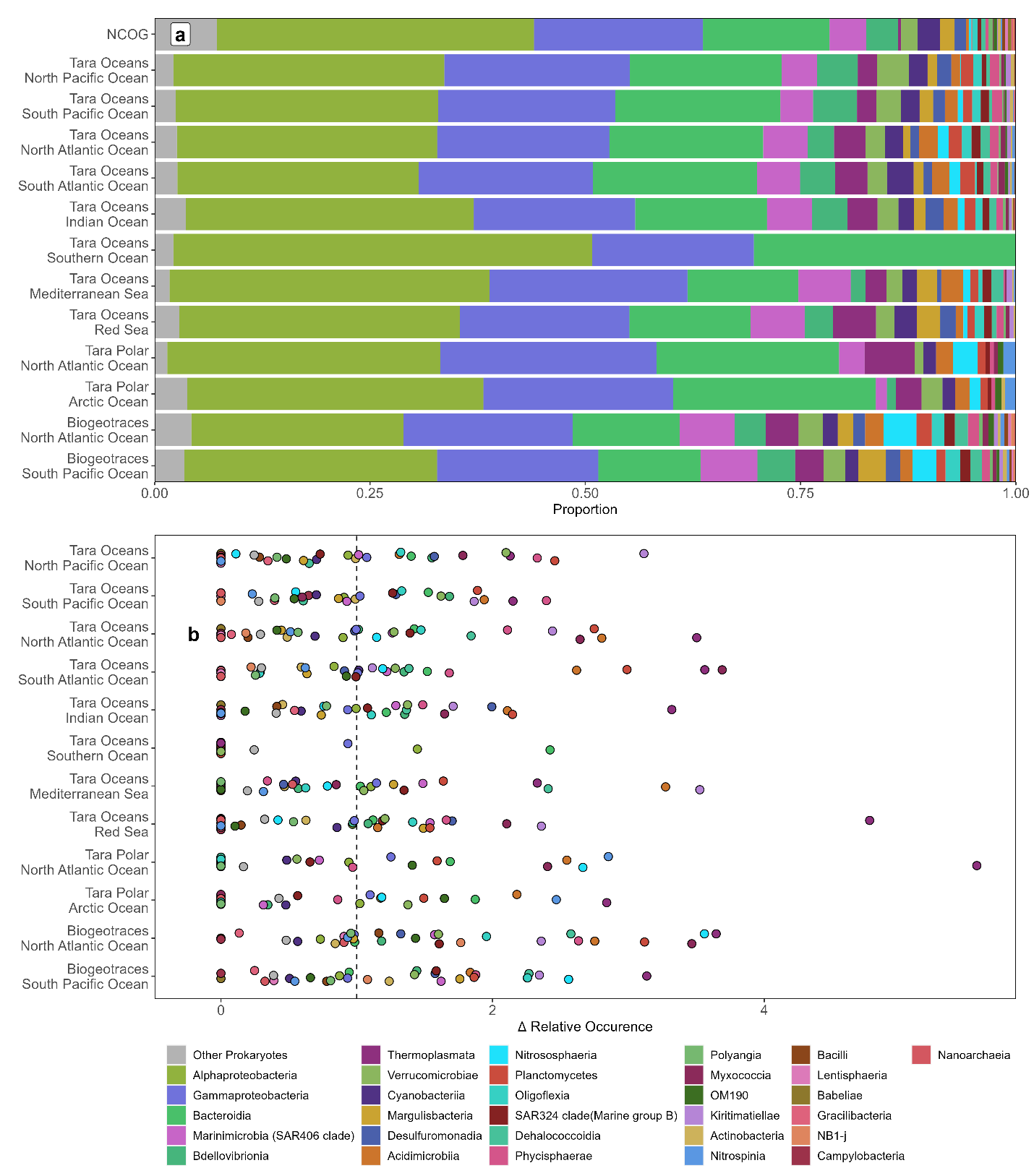


**Supplementary** **Figure 3:** Taxonomic composition of 16S ASVs per region. **a**, Relative proportion of ASVs in broad taxonomic groups per regional dataset **b**, Representation of broad taxonomic groups (# ASVs) in each regional dataset relative to their representation across all NCOG 16S ASVs. Δ Relative Occurrence = Proportional Richness per region / Proportional Richness within NCOG per taxonomic group. Larger numbers indicate that a given taxonomic group represents a larger proportion of a particular regional richness compared to its proportional richness in NCOG. Zero values indicate that that taxonomic group is not found in a particular region.

**
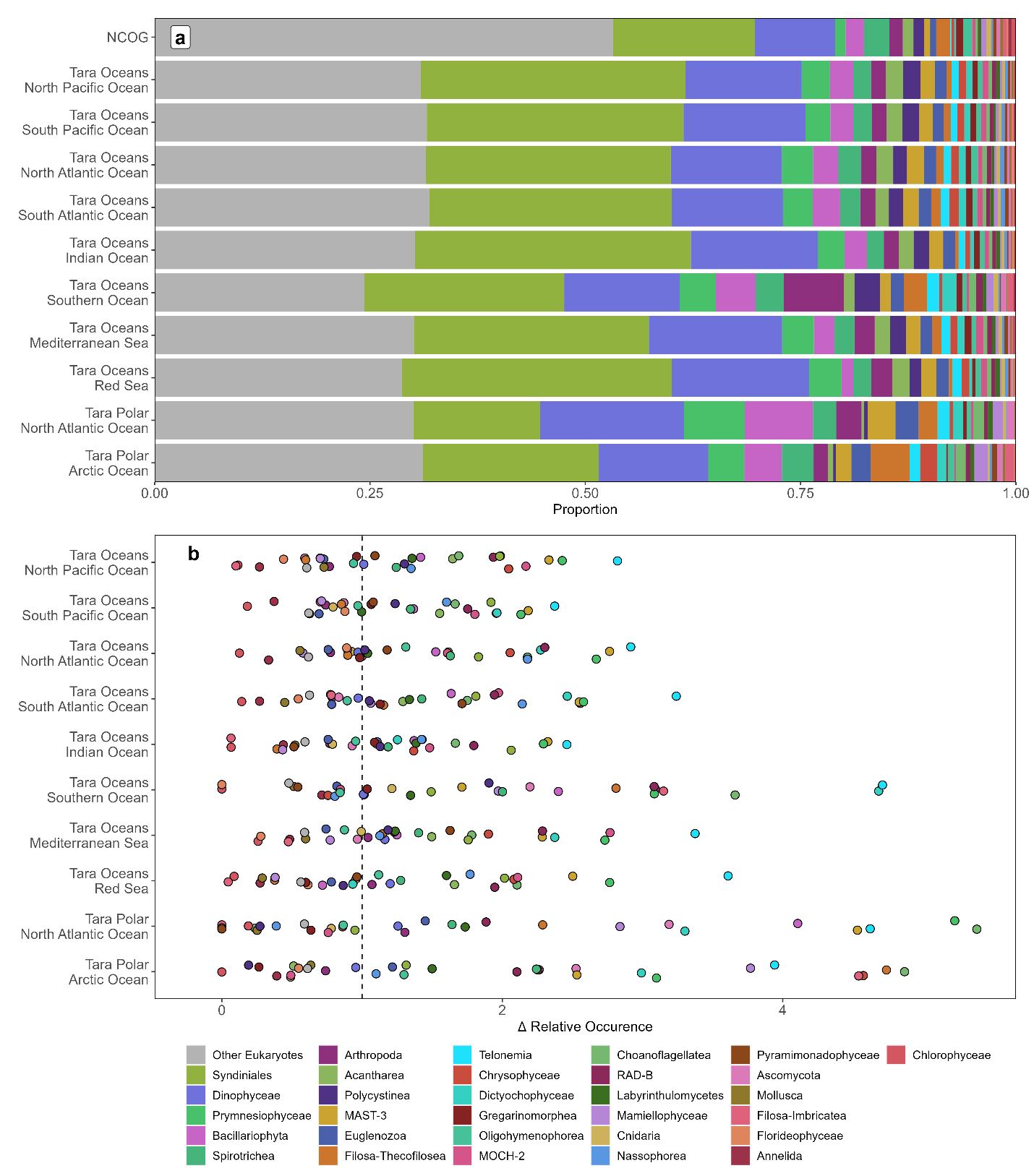
**

**Supplementary** **Figure 4:** Taxonomic composition of 18S-V9 ASVs per region. **a**, Relative proportion of ASVs in broad taxonomic groups per regional dataset **b**, Representation of broad taxonomic groups (# ASVs) in each regional dataset relative to their representation across all NCOG 18S-V9 ASVs. Δ Relative Occurrence = Proportional Richness per region / Proportional Richness within NCOG per taxonomic group. Larger numbers indicate that a given taxonomic group represents a larger proportion of a particular regional richness compared to its proportional richness in NCOG. Zero values indicate that that taxonomic group is not found in a particular region.
